# Surgically Discovered Xanthogranulomatous Pyelonephritis Invading Inferior Vena Cava with Coexisting Renal Cell Carcinoma

**DOI:** 10.1100/tsw.2009.6

**Published:** 2009-01-18

**Authors:** Henry M. Rosevear, Melissa M. Meier, Brian L. Gallagher, Fadi N. Joudi

**Affiliations:** ^1^ Department of Urology, University of Iowa, Iowa City, IA, USA; ^2^ Department of Pathology, University of Iowa, Iowa City, IA, USA

**Keywords:** renal cell carcinoma, diagnostic imaging, xanthogranulomatous pyelonephritis

## Abstract

Xanthogranulomatous pyelonephritis (XGP) is a chronic inflammatory process that results in replacement of renal and/or perirenal tissue with a diffuse infiltrate of inflammatory cells referred to as xanthoma cells. We present a case of a 49-year-old man with an incidentally discovered renal mass with inferior vena cava (IVC) thrombus, who was found intraoperatively to have a significant inflammatory process involving the posterior wall of his IVC and right renal vein consistent with XGP surrounding a focus of clear cell renal cell carcinoma in the midportion of his right kidney.

## INTRODUCTION

Xanthogranulomatous pyelonephritis (XGP) is an inflammatory process most often associated with a chronic infectious process involving *Proteus* bacteria[1]. In the last 4 decades, 15 cases of XGP associated with renal cell carcinoma (RCC) have been reported[2]. Renal or inferior vena cava (IVC) involvement of XGP has been reported seven times[3,4,5,6,7,8,9]. We report a case of XGP associated with RCC that also had renal vein and IVC involvement.

## CASE REPORT

A 49-year-old man with a 1-month history of worsening chest pain presented to the emergency room and was found to have elevated troponins, but no changes in his electrocardiogram. During workup of his chest pain, a CT angiogram found a 7- × 5-cm right-sided renal mass. The same CT showed multiple bilateral 1-cm spiculated pulmonary lesions suspicious for metastases. A follow-up CT 1 month later noted that the renal lesion had decreased in size to 5 × 4.5 cm. Three months later, after being treated for his coronary artery disease with stenting, he was evaluated in the urology clinic for consultation of his presumed metastatic renal cancer. At consultation, the patient was asymptomatic, specifically denying any abdominal pain, fever, or recurrent urinary tract infections. He reported one episode of gross hematuria 1 year prior, attributed to a kidney stone, although he did not have symptoms of renal colic and no imaging was done. He also reported a 40-lb unintentional weight loss over the last 6 months. A CT done at consultation showed that the renal lesion had decreased in size to 4 × 3.8 cm and that most, but not all, of the pulmonary lesions had resolved. There was no evidence of hydronephrosis, renal or ureteral calculi. A new 2.5- × 2-cm low-attenuation lesion in the right renal vein extending into the IVC consistent with thrombus was also found (Fig. 1). This was further confirmed by MRI (Fig. 2).

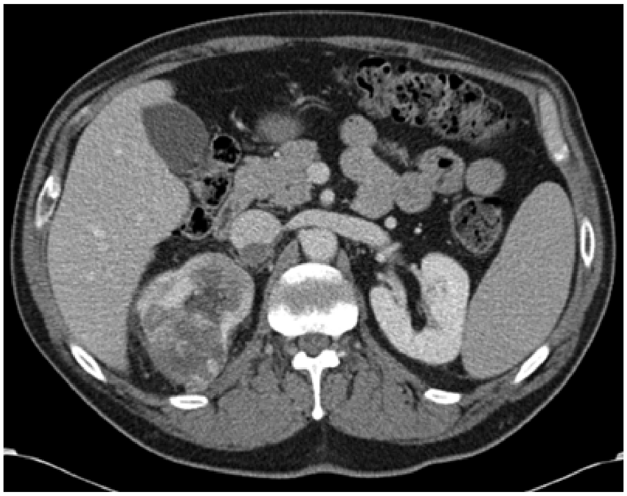

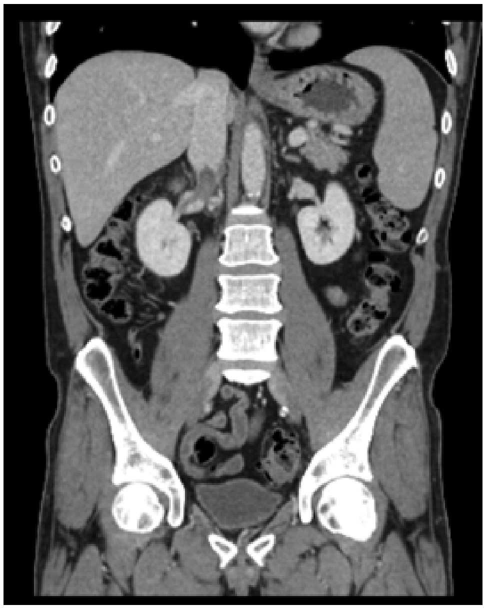

**FIGURE 1**. Axial (left) and coronal (right) CT images of the right kidney showing a midportion renal mass and tumor thrombus in the IVC.

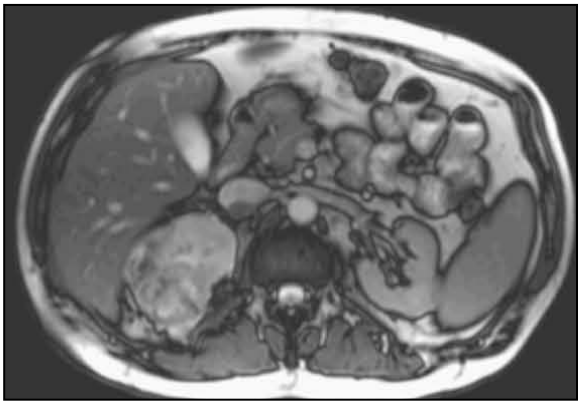

**FIGURE 2.** Axial MRI image of the right kidney demonstrating a mass in the midpole region and a thrombus in the IVC.

Despite the fact that the renal mass and pulmonary nodules decreased in size, it was felt that the new finding of a renal vein and IVC thrombus was concerning for RCC and warranted surgical exploration. Intraoperatively, a firm area was appreciated posterior to the IVC and right renal vein. No freely mobile thrombus in the IVC or renal vein could be palpated. Intraoperative ultrasound (US) was obtained, and it showed that the mass was adherent to the inner posterior wall of the renal vein and IVC and not freely mobile in either. The mass could be followed back by US to the right kidney. Vascular surgery was consulted and determined that, based on the location and extent of the IVC involvement, a large vascular graft would be necessary. A nephrectomy was performed next, leaving the IVC in place. Frozen section analysis revealed XGP in the mass involving the renal vein. After further discussion with vascular surgery colleagues, we elected to leave the IVC thrombus in place as it was felt to be an inflammatory mass that was densely adherent to the wall of the cava and replacing a segment of the IVC was not warranted, especially that doing so may have compromised the left renal vein. Final pathology also revealed a 3.7-cm clear cell RCC Fuhrman grade III confined to the kidney (pT1a, Figs. 3 and 4). The patient tolerated the procedure well and was discharged 3 days later after an uneventful recovery, well ahead of expectations given the extent of his surgery. We will follow this patient with serial CTs, the first of which was just completed at 6 months postsurgery and shows a stable mass at the IVC (Fig. 5). The exact nature of his pulmonary nodules remains unknown and they are decreasing in size. Given the early stage of his RCC and the decreasing size of the lesions, we believe these are infectious in nature, but will continue to monitor.

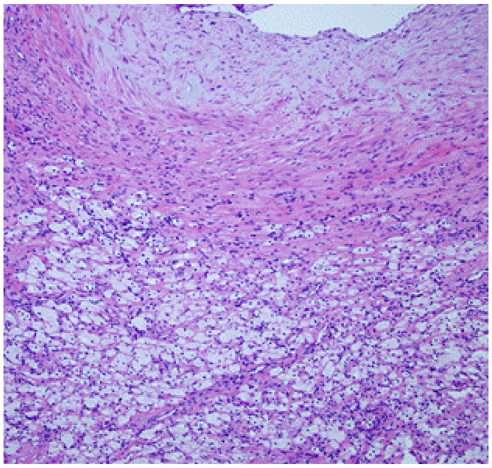

**FIGURE 3.** Histology showing XGP with characteristic xanthoma cells present surrounding and invading the IVC.

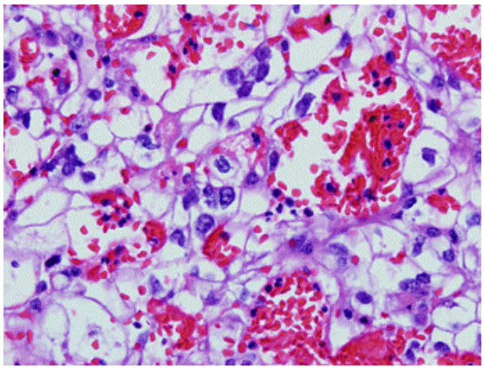

**FIGURE 4.** Histology showing conventional type clear cell RCC Fuhrman nuclear grade III with extensive vascularity.

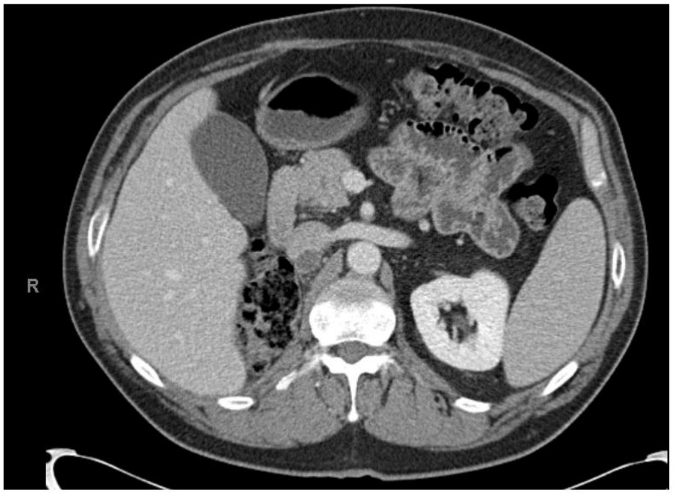

**FIGURE 5.** CT 6 months postsurgery showing a stable nonenhancing mass posterior to the IVC.

## DISCUSSION

To our knowledge, this is the first reported case of XGP associated with RCC that also had renal vein and IVC involvement. RCC can extend into the renal vein and in these cases, the thrombus is usually described as freely mobile[10]. It is unclear from the literature whether XGP involving the renal vein or the IVC creates a freely mobile thrombus. Even in this modern era of imaging where US, CT, and MRI are available, it can be difficult to distinguish XGP from RCC preoperatively. Previous authors have cited hydronephrosis, stone presence or a history thereof, and parenchymal thickening as possible hints to differentiate XGP from RCC[11,12,13,14]. In this case, the absence of renal calculi and the presence of a thrombus made the diagnosis more challenging. Of note, the thrombus was indeed an inflammatory mass that involved the wall of the renal vein and IVC. Clues that this may have been an inflammatory process include the decrease in size of the renal mass as well as the intraoperative finding of a thrombus that extended into and was not separate from the wall of the renal vein and IVC. Further, because of the multiple CTs that were taken over a course of 4 months, the rapid progression to IVC involvement in a period of 1 month could be indicative of an inflammatory process. The preoperative MRI allowed us to further define the extent of the thrombus, which can be important for surgical planning, but was not helpful in modifying the presumed presurgical diagnosis of renal malignancy with tumor thrombus.

XGP can be classified as either focal or diffuse forms with the diffuse form more common (85%)[14]. The focal or segmental form is more often confused with other pathologies because it often lacks the hydronephrosis or stone burden that can be a clue towards its correct diagnosis. Ruling out renal cancer in this case would not have been possible preoperatively, and surgical exploration was prudent. This rare case highlights the need for careful intraoperative evaluation of unexpected findings as well as serving as a reminder of the need to include XGP in the differential diagnosis of RCC. In the era of modern imaging, XGP remains a "great imitator" of RCC, even in the setting of renal vein and IVC thrombus.
